# Report of two rare cases of adrenal incidentalomas with different origins: revisiting pathological and radiological findings with a short review of the literature

**DOI:** 10.1186/s43046-020-00039-z

**Published:** 2020-05-27

**Authors:** M. A. Elbaset, Mohamad H. Zahran, Mohamed Badawy, M. Abd Elhameed, Yasser Osman

**Affiliations:** 1 Urology Department, Urology and Nephrology Center, Mansoura University, Mansoura, Egypt; 2 Radiology Department, Urology and Nephrology Center, Mansoura University, Mansoura, Egypt; 3 Pathology Department, Urology and Nephrology Center, Mansoura University, Mansoura, Egypt

**Keywords:** Adrenal, Tumors, Non-Hodgkin’s lymphoma, Angiosarcoma

## Abstract

**Background:**

Adrenal tumors can be detected incidentally in 4 to 8% of patients radiologically. Adenomas, pheochromocytomas, and adrenocortical carcinomas represent the most common tumors of the adrenal glands. Rare histopathological findings are uncommon. We aimed to report two rare primary adrenal tumors diagnosed initially as incidentalomas to identify clinical characteristics, management, and clinical outcomes after treatment.

**Case presentation:**

The first case was a 52-year-old man presented with an incidentally discovered locally advanced primary adrenal angiosarcoma. The patient was managed surgically with no adjuvant therapy. The patient was followed up for 3 years without evidence of local recurrence. The second case was a 63-year-old woman, presented with an incidentally discovered primary diffuse B-cell lymphoma of the left adrenal gland. She was treated by adrenalectomy. Later on, adjuvant six cycles of CHOP (cyclophosphamide, doxorubicin, vincristine, and prednisolone) chemotherapy were given. After 6 months follow-up, the patient was alive and disease-free.

**Conclusion:**

The diagnosis of adrenal tumors increased nowadays because of the widespread use of imaging studies, though rare pathologies should be taken into consideration.

## Background

Different tumors with different origins can be detected among adrenal neoplasms. Tumors such as adrenocortical adenomas and carcinomas represent the most common tumors arising from the adrenal cortex [1]. On the other hand, the adrenal medulla is a common site for pheochromocytoma and neuroblastic tumors. Moreover, vascular tumors as angiosarcoma were reported to arise primarily from the adrenal gland in a few cases [2, 3]. Angiosarcoma represents less than 2% of soft-tissue sarcomas [4]. They commonly occur in the breast, skin, spleen, bone, and liver [4].

Primary adrenal lymphoma without extra-adrenal involvement is extremely rare (less than 1%) [5–7]. Secondary adrenal lymphoma usually occurs with widespread or advanced stages of the disease, with mortality reaching 18 to 25% [8, 9].

Being rare pathological entities, we reported here two cases of incidentally diagnosed primary adrenal angiosarcoma and diffuse B-cell non-Hodgkin’s lymphoma with a detailed description for clinical, histological features and outcomes.

## Case presentation

### Case No. 1

**Fig. 1:**
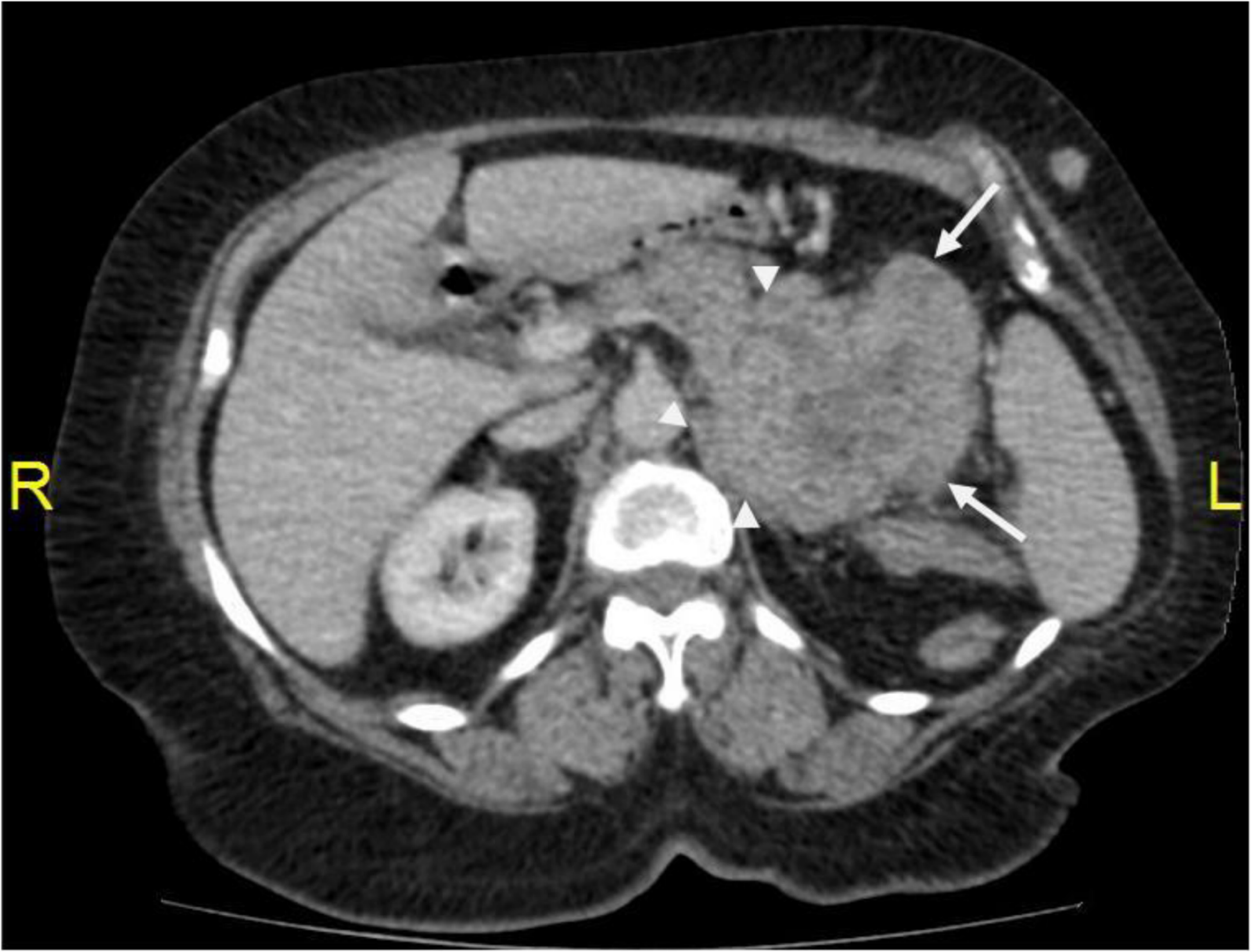
Contrast-enhanced CT scan of the abdomen showing large ill-defined soft tissue mass arising from the body and lateral limb of left suprarenal gland (arrows), and the mass exhibits heterogeneous postcontrast enhancement with areas of cystic degeneration, and it was seen inseparable from the body and tail of the pancreas as well as the left diaphragmatic crus (arrow heads)

A 52-year-old hypertensive man presented with incidentally detected left adrenal mass. All metabolic adrenal workup (24 h urinary cortisol, urinary metanephrines, serum aldosterone, and serum K^+^ level) were normal. Contrast-enhanced computed tomography (CECT) was done and showed left non-adenomatous heterogeneous adrenal mass 7 × 9 cm (Fig. 1). Bone scan was carried out owing to high serum alkaline phosphatase and was free. The patient was managed by open adrenalectomy via the thoracoabdominal approach. The mass was adherent to the diaphragm, the tail of the pancreas, and the upper pole of the left kidney. Total excision was done completely with difficulty. Gross examination of the specimen revealed a rounded mass measured 11 × 9 × 7 cm, firm in consistency with a thickened whitish capsule. Cut section (C/S) showed variegated appearance and alternating grayish and yellowish colored areas with dark red areas of hemorrhage. Microscopic examination (M/E**)** showed infiltration by atypical anastomosing vascular spaces lined by endothelial cells exhibiting large vesicular nuclei and abundant eosinophilic cytoplasm. Abnormal mitotic figures were seen 9–19/10 HPF. Sheets like areas were also seen which lacked the vasoformative architecture.

**Fig. 2:**
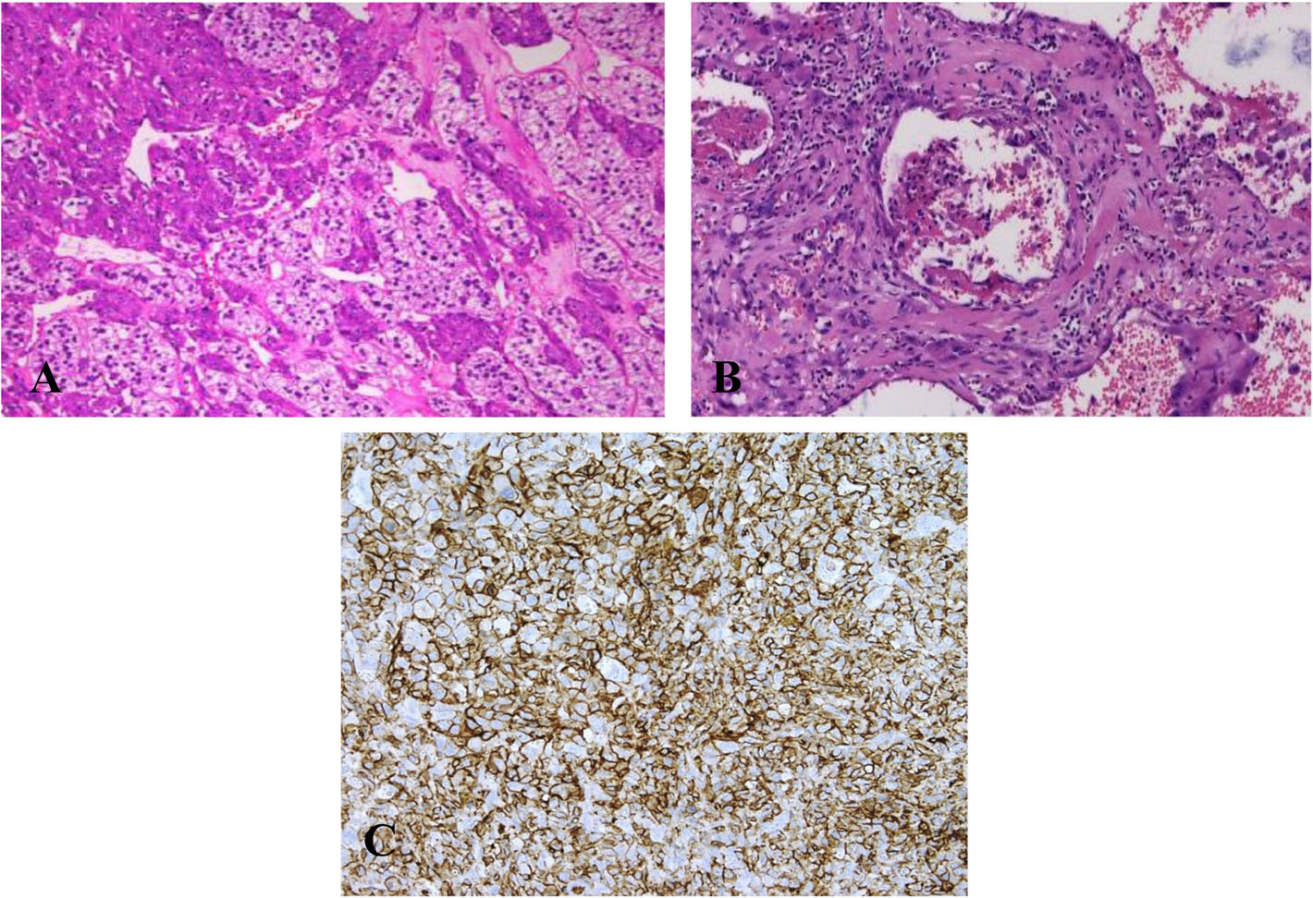
**a** Primary adrenal angiosarcoma showing irregular anastomosing vascular channels lined by a typical pleomorphic cells infiltrating the adrenal cortical cells (Hematoxylin and eosin, × 100). **b** Infiltration by atypical anastomosing vascular spaces lined by endothelial cells exhibiting large vesicular nuclei and abundant esoinophilic cytoplasm were seen (Hematoxylin and eosin, × 200). **c** Immunohistochemical staininsg showed diffuse intense membranous reaction in tumor cells for CD 31 (× 100)

Extensive necrosis was also noted. Immunohistochemical staining for CD31 showed a diffuse intense membranous reaction in tumor cells (Fig. 2). The case was diagnosed as primary adrenal angiosarcoma. The patient did not receive any adjuvant therapy postoperatively. Last follow-up CT and bone scan were 3 years later with no evidence of local or distant recurrence.

### Case No. 2

**Fig. 3:**
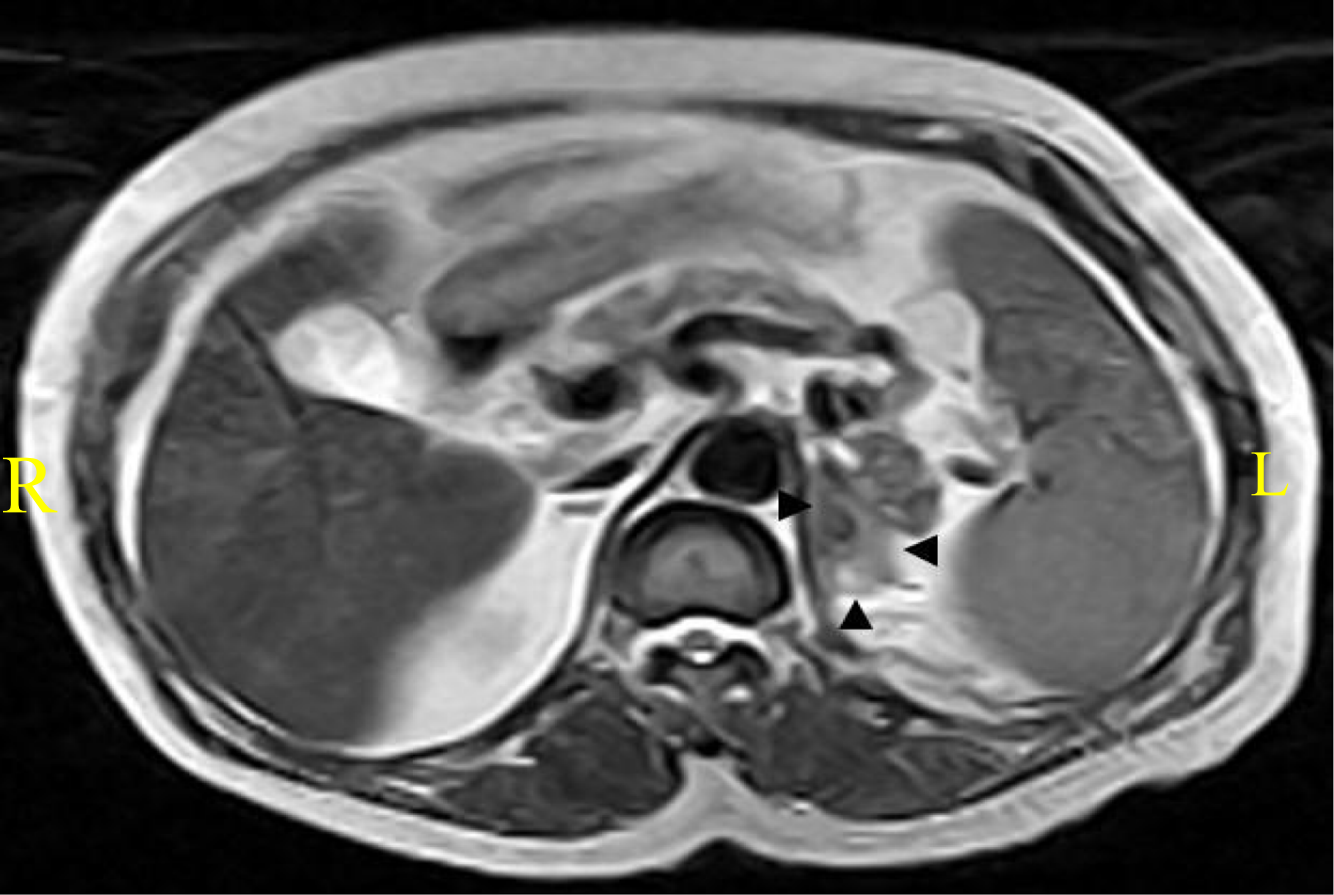
Axial T2WI MRI of the abdomen showing micro-lobulated soft tissue lesion arising from left suprarenal gland (arrow heads) displaying heterogeneous SI at T2WI

**Fig. 4:**
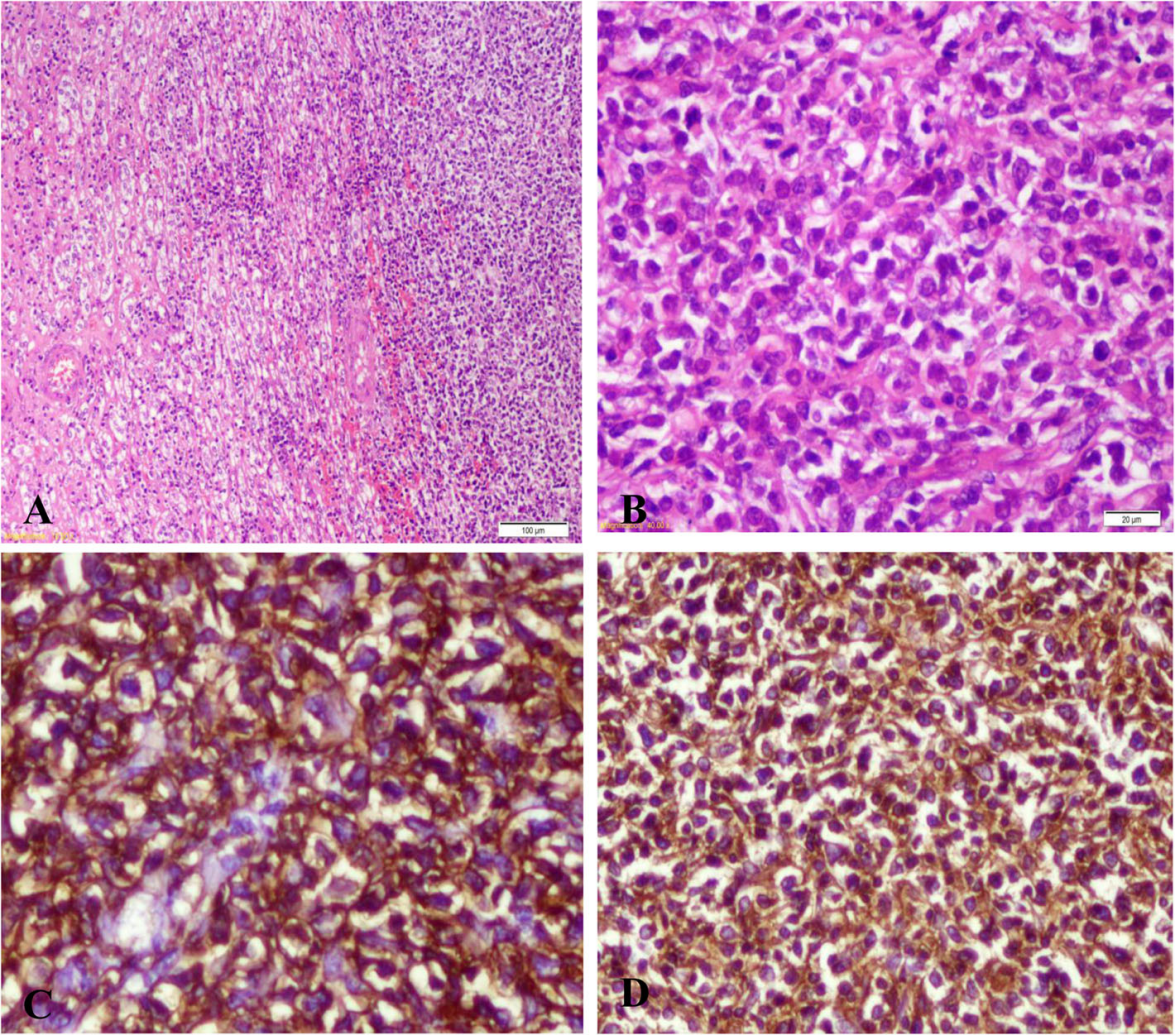
**a** Primary adrenal lymphoma showing infiltration of the adrenal cortex by diffuse proliferation of large transformed B lymphocytes (Hematoxylin and eosin, × 100). **b** The cells showed enlarged nuclei, conspicuous nucleoli and scanty cytoplasm with scattered mitotic figures (Hematoxylin and eosin, × 400). **c**, **d** Diffuse membranous staining in transformed lymphocytes for CD20 and LCA, respectively (× 200)

A 63-year-old woman presented with an incidentally discovered left adrenal mass. The patient suffered from uncontrolled type II diabetes mellitus and hypertension. She was maintained on two different antihypertensive drugs (calcium channel blocker and beta-blocker). No signs of immunodeficiency were identified. All laboratory investigations were within normal values in addition to normal metabolic adrenal workup. MRI revealed left non-adenomatous adrenal mass measuring 4 × 3 × 3 cm with no other organomegaly (Fig. 3). The patient was managed by open left adrenalectomy. The mass was locally advanced, encasing both left renal vein and renal artery with close adherence to the aorta. Excision of the mass was done along with the excision of multiple associated lymph nodes around the aorta and renal pedicle. Grossly, there was firm enlarged adrenal gland measured 4 × 3 cm. The C/S revealed yellowish white mass replacing the whole gland with multiple surrounding lymph nodes which have grayish-white solid homogenous C/S; the largest measured 3 × 3 cm.. Microscopically, there was infiltration of the adrenal cortex by diffuse proliferation of large transformed B lymphocytes with enlarged nuclei, conspicuous nucleoli, and scanty cytoplasm. Abnormal mitotic figures were seen 5–20/10 HPF. The tumor was associated with focal areas of necrosis. Dissected lymph nodes were tumor-free. Immunohistochemical studies for LCA and CD-20 showed a diffuse membranous reaction in transformed lymphocytes, while CK, inhibin, and HHV8 were negative. Also, immunostaining for CD-10 and BCL-6 were negative. The final diagnosis was non-germinal center diffuse large B-cell lymphoma of the adrenal gland (Fig. 4). The patient was referred to the oncology center for adjuvant chemotherapy (cyclophosphamide, doxorubicin, vincristine, and prednisolone) (CHOP protocol). She received 6 cycles of chemotherapy. Follow-up MRI six months later showed no evidence of local recurrence or distant metastasis.

## Discussion

Adrenal incidentaloma (AI) poses a diagnostic challenge in part due to its rarity, whereas radiological studies are the cornerstone for diagnosis [10]. Most of AIs are benign tumors, but a careful evaluation is required to rule out malignancy and functioning adenomas. Primary adrenal angiosarcoma and non-Hodgkin’s lymphoma are uncommon adrenal lesions; therefore, diagnosis and management are still a matter of debate till now [10–12].

*Primary adrenal angiosarcoma* is challenging to the clinician because of its scarcity [11]. Besides, the lesion is usually masked by concomitant necrosis and hemorrhage adding more difficulty in diagnosis. In a recent case reports, the incidence of adrenal angiosarcoma is more common among males, especially in the fifth and sixth decades of life [12, 13]. Likewise, our patient’s age and gender were matched with previously documented data. The clinical presentations are variable; some cases are asymptomatic, and others can show non-specific complaints as abdominal pain, weight loss, and fever [12, 13]. According to our findings, the patient was asymptomatic with an incidentally discovered adrenal mass during periodic follow-up. Grossly, the tumor may be predominantly cystic. Microscopically, adrenal angiosarcomas are frequently characterized by epithelioid appearance lacking the vasoformative patterns. These tumors typically stain positive for cytokeratin, an epithelial tumor marker, which can be seen in metastatic epithelial tumors or other mesenchymal neoplasms [3]. Cells are large and round with prominent nucleoli, while nuclei may appear vesicular [14]. Pathological examination for the excised adrenal mass in our case showed typically the previous reported pathological findings. Alternatively, accurate diagnosis by imaging is quite challenging, as there are no pathognomonic findings [3]. CECT images demonstrate heterogeneous low attenuation suggesting tumor necrosis. Whereas hyper attenuation suggests hemorrhage or calcification, postcontrast images may reveal heterogeneous enhancement and areas of necrosis [13]. CECT of the abdomen in our case showed large ill-defined soft tissue mass arising from the body and lateral limb of left suprarenal gland, and the mass exhibited heterogeneous post-contrast enhancement with areas of cystic degeneration and necrosis.

There is no doubt that adrenalectomy serves both diagnostic and therapeutic purposes for angiosarcoma. Almost all cases could be managed by adrenalectomy alone. On the other hand, postadrenalectomy treatment is still controversial. A multi-modal treatment approach was previously adopted and included postoperative doxorubicin-based chemotherapeutic regimens and adjuvant radiation therapy (XRT) [12]. Fleutra et al. discussed forty reported cases of primary adrenal angiosarcoma managed by different approaches of treatment. Twenty-two patients (55%) were managed by adrenalectomy only without concomitant adjuvant or neoadjuvant therapies. Of them, five patients (22.7%) were manifested by either local recurrence or distant metastasis at median (range) 21 (6–24) months, and eight patients (36.4%) were disease-free at follow-up at median (range) 59 (6–144) months. The remaining patients (40.9%) either died postoperatively or had not follow-up data [11]. In our report, the patient was managed by wide surgical excision only without adjuvant treatment. After 36 months, the patient was alive and disease-free.

*Primary adrenal lymphoma* is presented commonly in the elderly with a mean age of 62 years at presentation except in some rare reports [15]. The adrenal glands are involved in 24% of patients with multi-organ lymphoma [16]. Isolated unilateral primary adrenal lymphoma is very rare and constitutes 1% of extra-nodal lymphoma [16, 17]. The most common subtype found in the adrenal glands is diffuse large B-cell lymphoma (DLBCL) [16]. Flank or abdominal pain and fatigue are considered the most presenting symptoms, and only 1% of tumors were detected incidentally. Associated skin hyperpigmentation, organomegaly, and lymphadenopathy were presented in 27%, 15%, and 7%, respectively [7]. By MRI, primary adrenal lymphoma is characterized by isointense or hypo intense lesions in T1-weighted images and hyperintense lesions in T2-weighted images [18]. On diffusion-weighted imaging (DWI), lymphomas usually generate restricted diffusion and high signal intensity on DWI due to high cellularity of the tumor [19]. Prognostic factors including age, adrenal insufficiency, and tumor size have a significant impact on treatment outcomes and survival. Our case diagnosed as of non-germinal center origin with negative staining for CD-10 and BCL-6. Adjuvant chemotherapy is given to prevent disease recurrence (CHOP/CHOP-like) and regimens are the most commonly administered chemotherapy protocols to treat the primary adrenal lymphoma [20]. Also, the prognosis has been slightly improved with the recent use of rituximab as a new chemotherapeutic agent [16, 21]. Although a median survival of nearly 3 months was previously reported [21], the most recent data suggest disease-free survival of 12 months [16]. Our case was a 63-year-old female presented with an incidentally diagnosed isolated unilateral adrenal lesion with no signs of immune deficiency (excluding HIV infection with also negative staining for HHV8). Heterogeneous hyperintense signal intensity at T2WI was a characteristic sign in MRI. Postadrenalectomy, adjuvant six cycles of chemotherapy (CHOP protocol) were given to the patient. After six months of follow-up, the patient was disease-free.

## Conclusion

Primary adrenal angiosarcoma and lymphoma are of rare entities carrying a prodigious challenge in diagnosis and management. Both tumors could be presented as an incidentalomas. A multidisciplinary approach is of value in such cases for proper management.

## Data Availability

The datasets used and/or analyzed during the current study are available from the corresponding author on reasonable request.

## Abbreviations

C/S: Cut section
MRI: Magnetic resonance imaging
CECT: Contrast-enhanced computed tomography
